# Long-term persistence of piscine orthoreovirus-1 (PRV-1) infection during the pre-smolt stages of Atlantic salmon in freshwater

**DOI:** 10.1186/s13567-023-01201-w

**Published:** 2023-08-29

**Authors:** Dhamotharan Kannimuthu, HyeongJin Roh, Ma. Michelle D. Peñaranda, Øystein Wessel, Stig Mæhle, Ghebretnsae Dawit Berhe, Joachim Nordbø, Bjørn Olav Kvamme, H. Craig Morton, Søren Grove

**Affiliations:** 1 Institute of Marine Research, Nordnes, P.O. Box 1870, N-5817 Bergen, Norway; 2 Faculty of Veterinary Medicine, Norwegian University of Life Sciences, 1433 Ås, Norway

**Keywords:** PRV-1, salmon fry, persistence, stress, HSMI, erythrocytes, shedding

## Abstract

Piscine orthoreovirus (PRV) causes heart and skeletal muscle inflammation (HSMI) in farmed Atlantic salmon. During salmon production cycles, HSMI has predominantly been observed after seawater transfer. More recently, better surveillance and longitudinal studies have detected occurrences of PRV-1 in freshwater broodstock farms and hatcheries. However, very little is known about the viral kinetics of PRV-1 or disease development of HSMI during these pre-smolt stages. In this study, we conducted a long-term PRV-1 challenge experiment to examine the profile of viral load, infectiousness and/or clearance in Atlantic salmon during their development from fry to parr stage. Atlantic salmon fry (mean weight: 1.1 ± 0.19 g) were infected with PRV-1 (high virulent variant) via intraperitoneal (IP) injection. The viral load reached a peak at 2–4 weeks post-challenge (wpc) in heart and muscle tissues. The virus was detected at relatively high levels in whole blood, spleen, and head kidney tissues until 65 wpc. Heart and muscle lesions typical of HSMI were clearly observed at 6 and 8 wpc but then subsided afterwards resolving inflammation. Innate and adaptive immune responses were elicited during the early/acute phase but returned to basal levels during the persistent phase of infection. Despite achieving high viremia, PRV-1 infection failed to cause any mortality during the 65-week virus challenge period. Cohabitation of PRV-1 infected fish (10 and 31 wpc) with naïve Atlantic salmon fry resulted in very low or no infection. Moreover, repeated chasing stress exposures did not affect the viral load or shedding of PRV-1 at 26 and 44 wpc. The present findings provide knowledge about PRV-1 infection in juvenile salmon and highlight the importance of continued monitoring and management to prevent and mitigate the PRV-1 infection in freshwater facilities.

**Supplementary Information:**

The online version contains supplementary material available at 10.1186/s13567-023-01201-w.

## Introduction

Piscine orthoreovirus is a double stranded RNA virus that belongs to the family Spinareoviridae [1]. It is a non-enveloped spherical virus, measuring 70 nm in size, with inner and outer capsid layers [2]. Its genome consists of 10 segmented linear double stranded RNA, with sizes ranging from 1 kbp to 3.9 kbp [3]. Whole genome analysis and host species specificity have led to the classification of PRV into three genotypes, namely PRV-1, PRV-2, and -3. PRV-1 is responsible for heart and skeletal muscle inflammation (HSMI) in Atlantic salmon (*Salmo salar*) [2], while PRV-2 causes erythrocytic inclusion body syndrome (EIBS) in Japanese coho salmon (*Oncorhynchus kisutch*) [4]. Additionally, PRV-3 induces HSMI-like heart inflammation in rainbow trout (*Oncorhynchus mykiss*) [5]. Moreover, PRV-1 has been subcategorized into high and low virulent genotypes based on the whole genome and the recently published in vivo challenge study [6]. The high virulent PRV genotype is more prevalent and is the primary variant causing heart and skeletal muscle inflammation in farmed Atlantic salmon in Norway [6]. While the low virulent genotype prevalent in North America and Norway and is shown to cause mild HSMI in challenge experiments [6, 7]. In Norwegian aquaculture, HSMI causes severe pathological changes, high morbidity, and mortality up to 20% [8].

The pathogenesis and virulence differences of PRV-1 have been extensively studied in farmed Atlantic salmon through experimental infection and longitudinal studies [2, 8–10]. In the early phases of the infection, PRV-1 primarily infects erythrocytes, where it replicates in cytoplasmic viral inclusions [10–12]. Although PRV-1 causes transient reduction in blood hemoglobin, it does not result in anemia in Atlantic salmon [13]. During the peak of infection, the virus is released from infected erythrocytes and can be detected in plasma, subsequently leading to secondary infection in other target organs like heart, and muscle, causing epicarditis, myocarditis and myositis [14]. Antiviral immunological responses are dominated by the infiltration of CD8^+^ cells and are the primary cause of heart and muscle inflammation [15]. Nevertheless, direct virus-induced necrotic and degenerative changes in heart and muscle are also observed [2]. PRV-1 is eventually cleared from cardiomyocytes and myocytes, and resolution of inflammation occurs [14]. However, persistent PRV-1 infection with the low virulent genotype has been reported in Atlantic salmon post-smolts in Canada [16]. In rainbow trout, PRV-3 is shown to cause anemia and the virus is cleared from the infected fish after 10 weeks, indicating virus- and host-specific pathogenesis of PRV genotypes [17]. PRV-1 cannot be cultivated in cell lines, so neutralizing assays are not available for this virus. Nevertheless, studies utilizing bead-based assays have shown that fish infected with PRV-1 produce IgM antibodies against viral proteins, including sigma1, mu1c and muNS [18].

Fish erythrocytes are nucleated and have translation machinery that can support viral replication. Fish virus such as infectious salmon anemia virus (ISAV) hitchhike on erythrocytes by binding with little or no viral entry and replication before reaching the target tissues [19]. In contrast, PRV-1 infects and replicates in viral factories in the cytoplasm of erythrocytes before spreading to other secondary target organs [14, 20]. Several intrinsic and extrinsic factors can affect the number of erythrocytes in an individual fish [21]. Erythrocytes are continuously replenished by hematopoietic tissues in head kidney. Fish erythrocytes are capable of producing interferons, interferon-stimulated antiviral genes, *MX1*, *RSAD2* (viperin), and other factors to combat viral infections [22]. Infected and senescent erythrocytes are scavenged and eliminated from circulation by macrophages [23].

Compared to open sea pens, strict biosecurity measures can be implemented in freshwater Atlantic salmon smolt facilities. In addition, smolts are vaccinated prior to seawater transfer for selected pathogens to prevent disease outbreaks in pens. Nevertheless, viral disease outbreaks and increased mortality are common after seawater transfer. HSMI has been observed in freshwater smolt facilities without high mortality, but clinical disease outbreaks can occur as early as 14 days after seawater transfer [8]. Currently, there is no commercially available vaccine for PRV-1. Longitudinal studies in Atlantic salmon farms for HSMI, CMS, and PD disease outbreaks indicate that a clinical disease outbreak can persist for several months after the first detection of virus [8, 24, 25]. However, it is not known whether it is due to persistent infection of individual fish or due to prolonged virus shedding and transmission.

Although RNA viruses typically cause acute infections, recent studies have reported an increase in the incidence of persistent RNA virus infections [26]. In farmed Atlantic salmon, it has been shown that fish that survive infection with infectious pancreatic necrosis virus (IPNV) or salmon pancreas disease virus (SPDV) can become long-term carriers [27]. In addition, stress-mediated re-activation of viral infection followed by disease outbreaks have been reported for infectious pancreatic necrosis (IPN) [28], and salmon pancreas disease (SPD) [25]. After acute virus infection and disease recovery, some viruses can establish latent infection in specific tissues or cells, including immune privileged sites such as nerve tissues, brain, retina, gonads, etc. However, the presence of viral RNA without detection of infectious virions has also been shown for some viruses [26].

We have limited knowledge on the spatial and temporal viral dynamics of PRV-1 kinetics in the pre-smolt stages of Atlantic salmon. The aims of this study were to investigate the following aspects in experimentally challenged Atlantic salmon with PRV-1, (i) the spatiotemporal kinetics of PRV-1 viral load, (ii) expression of selected innate and adaptive immune genes, and (iii) histopathological changes in experimentally challenged Atlantic salmon. In addition, we examined the infectiousness of PRV-1 infected fish at selected time points, and also the effect of fright induced stress on viral load.

## Materials and methods

### Experimental fish

Atlantic salmon fry (StofnFiskur, Iceland) used in this experiment were procured from the Industrial and Aquatic Laboratory (iLab), Bergen. The fish were acclimatized in three 400 L freshwater tanks for two weeks before the start of the experiment. At the start of the challenge, the mean weight of the salmon fry was 1.1 ± 0.19 g (mean ± SD). Fish were tested by TaqMan RT-qPCR method, and confirmed negative for piscine orthoreovirus (PRV), infectious pancreatic necrosis virus (IPNV) [29], infectious salmon anemia virus (ISAV) [30], salmonid alphavirus (SAV) [31], and piscine myocarditis virus (PMCV) [32]. The water temperature was maintained at 10 to 12 °C throughout the experimental period. The fish were maintained on a 12:12 h light dark regime. All the experimental procedures were reviewed and approved by the Norwegian Animal Research Authority prior to start of challenge experiments (FOTS ID: 27105).

### In vivo virus propagation

Atlantic salmon post smolts, 1000–1100 g (*n* = 30) were intraperitoneal (IP) challenged with 100 µL of PRV-1 inoculum. The viral inoculum was prepared from PRV-1 infected blood (Ct 16.8) from fish challenged with the high virulent variant NOR-2018-NL [7]. Briefly, the blood cell pellet was diluted in L15 medium (1:10) and sonicated eight times at 20 kHz for 10 s on ice with 30 s intervals before being centrifuged at 5000 *g* for 5 min at 4 °C. The supernatant was used for the intraperitoneal challenging. A negative control inoculum was prepared in the same way from a blood cell pellet isolated from a noninfected fish.

To harvest the blood at the peak of viral load, 100 µL of blood was sampled weekly from the caudal vein of three random fish to estimate the viral load. At 4 weeks post-challenge, all the fish were euthanized, and the blood was collected in heparin tubes, separated plasma and blood pellet were stored at −80 °C (see Additional file 1).

### Experimental setup and sampling

**Figure 1:**
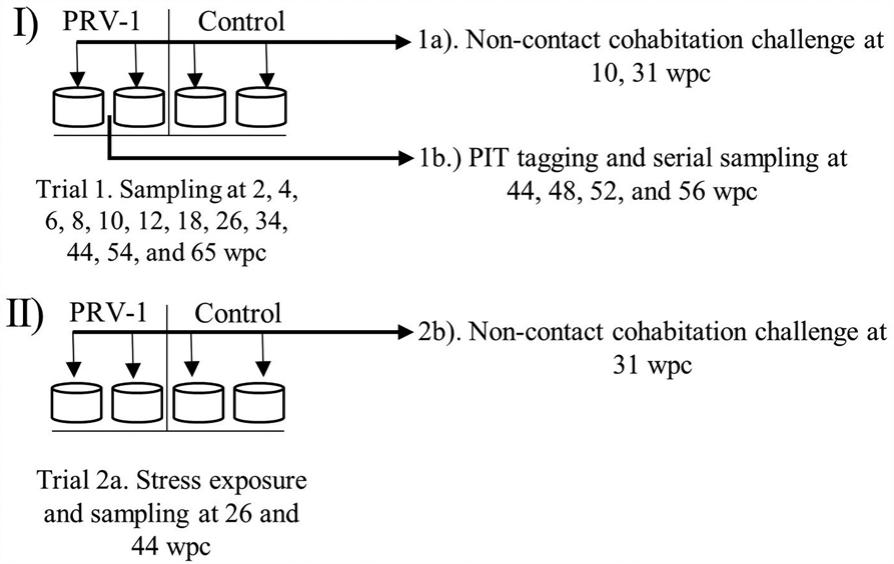
**Overview of experimental setup**.

All the infection experiments were conducted in the fish disease research facility at IMR, Bergen. The experiment was conducted in identical 250 L tanks and the water flow was maintained at 550 L/h. The experiment included two PRV-1-infected fish sampling tanks (*n* = 2 × 548), two PRV-1-infected fish observation tanks (*n* = 2 × 200), two non-infected fish sampling tanks (*n* = 2 × 182), and two non-infected observation tanks (*n* = 2 × 110) (Figure 1). Using 0.5 mL insulin syringes and 29G needle, the salmon fry were IP challenged with 10 µL of virus inoculum prepared from a blood cell pellet (Ct 15.38 diluted 1:10 in L15) isolated from PRV-1 infected fish from the above in vivo propagation. The non-infected controls were injected with 10 µL inoculum from uninfected fish. Fish were subsequently sampled at 2, 4, 6, 8, 10, 12, 18, 26, 34, 44, 54, and 65 weeks post-challenge (wpc). At each sampling, 16 fish from the infected groups (8 from each tank) and eight fish from the control groups (4 from each tank) were sampled for qPCR. In addition, 16 fish from the infected groups and eight fish from the control groups were sampled for histology. From 2 to 8 wpc, the whole organ package (all the internal organs from esophagus to anus), heart, and muscle were sampled separately for qPCR, and whole fish were sampled for histology. From 10 wpc, heart, spleen, head kidney, and muscle were sampled separately for histology and qPCR. From 18 wpc, a blood sample was also taken.

### Fright stress exposure experiments

Acute stress exposures can cause physiological alterations characterized by a transient surge in cortisol, glucose, heat shock proteins, hematocrit, hemoglobin levels, consequently impacting, immunocompetence, and growth. To determine the effect of fright stress on PRV-1 viral load, PRV-1 infected and control fish in two separate 150 L tanks (*n* = 2 × 242) were exposed to stress at 26 wpc and 44 wpc as demonstrated in a previous study [33]). Briefly, stress was created by reducing the water level in the experimental tanks to 50%, followed by chasing the fish continuously for 15 min with a net at moderate speed. This stress exposure was done every day for a period of seven days. Heart and spleen samples were taken day 0 (before stress), day 8 (one day post stress) and day 14 (seven days post stress) for viral load estimation.

### Cohabitation challenge experiments

To investigate whether persistently infected fish are able to transmit the virus, a non-contact cohabitation challenge experiment was conducted at 10 wpc and 31 wpc. The naïve salmon fry (0.5 g) of same strain as used in the main experiment were purchased from iLab for this experiment. In the first cohabitation experiment, naïve salmon fry (*n* = 2 × 32) were added to two small closed perforated tanks (Everdure Rotisseri, Heston Blumenthal). Each small fry tank was then immersed into a 250 L tank containing PRV-1 infected fish and cohabitated for 24 h (refer to Figure 4A), and after that the fry were moved to 3.5 L flow-through tanks (80-ZB30TK, Techniplast), and were maintained for the remainder of the experimental period (6 weeks). In the second cohabitation experiment at 31 wpc, to increase the cohabitation duration, custom-made tanks were designed to separate the shedder fish from naïve fry (Figure 4B). This separation prevented cannibalism and allowed us to feed the fish and hence maintain them together for a longer period without compromising their welfare. The water from the shedder (*n* = 15) compartment of the tank was set to flow through the cohabitant fry (*n* = 30) compartment. From both experiments, the organ package, heart, and muscle were sampled for the viral load estimation.

### PIT tagging and serial blood sampling

At week 44 post-infection, nine infected salmon with mean weight of 75 g, length 20 cm, were moved to a separate 150 L tank and PIT tagged for serial blood sampling. Fish were anesthetized and approximately 75 µL blood was collected from the caudal vein with 0.5 mL BD Microfine + 29 G needles every 4 weeks from 44 to 56 wpc.

### RNA extraction and RT-qPCR

Total RNA extraction from organ package, whole blood, blood cell pellet, heart, spleen, head kidney and muscle tissues were done with Promega Reliaprep simplyRNA HT 384 (Nerliens) on a Biomek 4000 Laboratory Automated Workstation (Beckman Coulter) according to the manufacturer’s instructions. RNA concentration was quantified using a NanoDrop™-1000 spectrophotometer (Thermo Fisher Scientific). The samples were normalized to a concentration of 25 or 50 ng/µL using the Biomek 4000 Laboratory Automated Workstation (Beckman Coulter). For double stranded RNA viruses like Rotavirus, Orthoreovirus, the double stranded RNA requires denaturation step at 95 °C for 5 min before RT. Hence, the total RNA was denatured by incubation at 95 °C for 5 min before used in a quantitative real-time RT-qPCR assay designed for targeting the PRV-1 segment S1 gene (Fwd TGCGTCCTGCGTATGGCACC, Rev—GGCTGGCATGCCCGAATAGCA and Probe FAM-ATCACAACGCCTACCT–MGBNFQ) [20]. However, skipping the denaturation step before RT results in less efficiency and amplifies mostly ssRNA as specified in a previous study [10]. To estimate viral replication, PRV-1 ssRNA was measured using total RNA without denaturation in the same quantitative real time RT-PCR assay. The sample and standard mixture was prepared using AgPath-ID One Step RT-PCR reagents (Thermo Fisher Scientific) according to the manufacturer’s instructions with RNA at a total concentration of 100 ng in a reaction mix containing 400 nM of forward primer, 400 nM of reverse primer, and 160 nM of probe in a total volume of 10 µL on a 384 well-plate. The Atlantic salmon elongation factor α 1 gene (EF1a) (Fwd CCCCTCCAGGACGTTTACAAA, Rev CACACGGCCCACAGGTACA and Probe FAM-ATCGGTGGTATTGGAAC–MGBNFQ) was used as endogenous control [34]. For the qPCR assay, amplification and fluorescence detection were performed on a QuantStudio™ 5 Real-Time PCR System (Applied Biosystems) for 40 cycles with a threshold value of 0.2 applied to all samples. Samples negative by qPCR were given a Ct value of 40 in figures and in statistical analyses.

### Histopathology

For histopathological analysis, whole fry (tail and fins removed) or hearts from parr were fixed in 4% formaldehyde solution, buffered pH 6.9 (Sigma-Aldrich) for 48 h and then transferred to 70% ethanol. After dehydration in a graded ethanol series and clearing with xylene in an Automatic Benchtop Tissue Processor (Leica BioSystems, Germany), the tissues were embedded in histowax. Sections were cut at 3 µm thickness and stained using Shandon instant hematoxylin (Thermo Scientific), erythrosine and saffron (Waldeck) (HES) staining. The sections were scanned using the NanoZoomer S60 digital slide scanner (Hamamatsu Photonics, United Kingdom) and the images were viewed using the software NanoZoomer Digital Pathology NDP.view2 (Hamamatsu Photonics). Selected sections were photographed using a Leica DMRBE microscope mounted with a SPOT Insight camera.

### Immune gene expression

For immune gene expression analysis, cDNA synthesis was done using SuperScript™ VILO™ cDNA Synthesis Kit (Invitrogen, Thermo Fisher Scientific) with either 250 ng (2, 4, 6 and 12 wpc) or 1000 ng (18 and 54 wpc) of total RNA from the heart of eight control and eight PRV infected fish. The real time qPCR assays for innate (*MX1*, *IL10*, and *IFNG*) and adaptive (*CD4*, *CD8a*8a, and *mIgM*) immune genes were setup with 0.5 or 10 ng cDNA equivalents of RNA, 400 nM forward and reverse primers, 29 nM reference dye, 2X Brilliant III Ultra-Fast SYBR® Green qPCR Master Mix. The reactions were run on QuantStudio™ thermal cycler (Applied Biosystems) and the results were visualized and analyzed using QuantStudio™ design and analysis software (v1.5.2). The qPCR conditions were as follows, 1 cycle of 95 °C for 3 min, 40 cycles of 95 °C for 5 s and 60 °C for 20 s, followed by melt curve analysis. The geometric mean values of housekeeping genes, elongation factor 1a (EF1a) and RNA polymerase 1 (RPL1) for each sample, were used as endogenous control for normalization. Primer sequences are given in Table 1. The relative expression of immune gene transcripts for all the infected groups were evaluated relative to the non-infected control groups as the calibrator at the same sampling time by the ΔΔCT method [35].

**Table 1:** **Overview of primers used in the study**.

Immune genes	Forward primer 5′–3′	Reverse primer 5′–3′	References
*EF1A*	CACCACCGGCCATCTGATCTACAA	TCAGCAGCCTCCTTCTCGAACTTC	[45]
*MX1*	GGTGGTTGTGCCATGCAA	TGGTCAGGATGCCTAATGTC	[46]
*IFNG*	GGTCCACTATAAGATCTCCAAGGA	CTGGCAAGATACTCCGATACAC	[46]
*IL10*	GCTATGGACAGCATCCTGAAGTT	GGTTGTTCTGCGTTCTGTTGTT	[47]
*CD4*	GTGGAGGTGCTACAGGTGTTTTC	GGGGAGGAGCCTAAAGCG	[48]
*CD8a*	CTTCAGCGAGGAGCAGATAAAC	GGCTGTGGTCATTGGTGTAGTC	[48]
*mIgM*	TGAGGAGAACTGTGGGCTACACT	TTAATGACTACTGAATGTGCAT	[47]
PRV-1	TGCGTCCTGCGTATGGCACC	R- GGCTGGCATGCCC GAATAGCA FAM-ATCACAACGCCTACCT– MGBNFQ)	[20]

### Statistical analysis

All the data visualization, graph preparation and statistical analysis were carried out with GraphPad prism 9 (v9.0.0). The differences in viral load and immune gene expression during the experiment were calculated based on one way ANOVA with Bonferroni multiple comparison test. Weight differences during the experiment between control and infected fish were compared with two-way ANOVA and Turkey multiple comparison test.

## Results

### PRV-1 persists in heart and muscle of Atlantic salmon fry until 65 wpc

**Figure 2:**
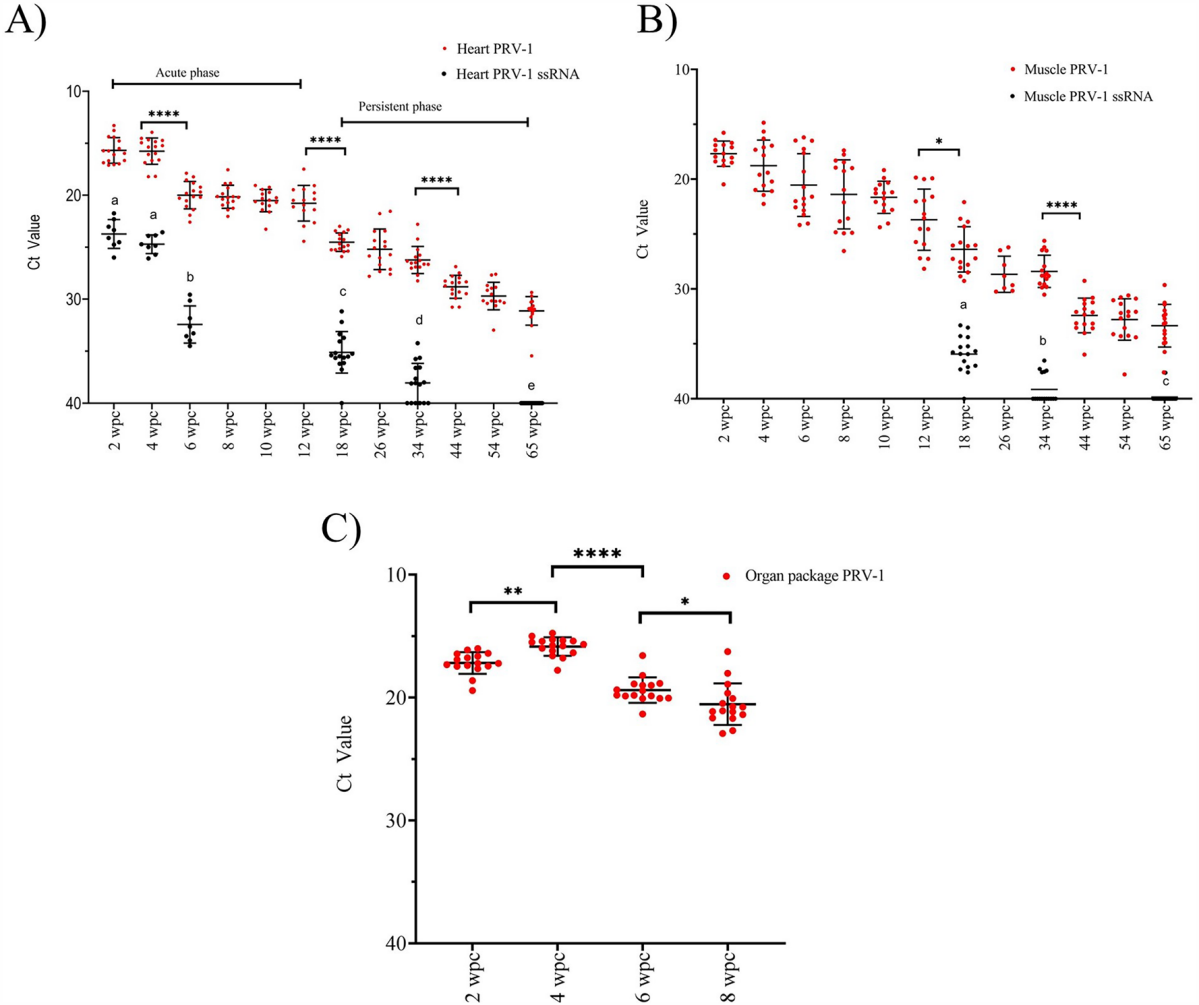
**The viral load of PRV-1 in the longitudinal challenge trial where salmon fry were IP challenged with PRV-1 and followed for 65 weeks**. The PRV-1 viral load in **A** Heart, **B** Muscle and **C** Organ package is indicated by red dots. PRV-1 single stranded RNA (ssRNA) is shown with black dots. Statistically significant differences are shown by combined bars and asterisks for total RNA; alphabetical letters for ssRNA. Asterisk (*P < 0.05; **P < 0.01; ***P < 0.001; ****P < 0.0001) indicate significant differences.

Organ packages from IP challenged fry were sampled from 2 to 8 wpc. The viral load in the organ packages peaked at 4 wpc and had significantly decreased (*p* < 0.0001) by 6 wpc (Figure 2).

In the heart, PRV-1 viral load reached its peak during week 2–4 with mean Ct values of 15.69 ± 1.2 and 15.77 ± 1.3, respectively, and then decreased significantly (*p* < 0.0001) at 6 wpc (Figure 2). Significant differences (*p* < 0.0001) in viral load were observed between 12 and 18 wpc, and between 34 and 44 wpc. The viral load at 65 wpc was Ct 31.1 ± 1.4. The ssRNA load peaked during 2- 4 wpc, had decreased significantly by 6 wpc and continued to decrease with significant reductions between all analyzed time points. At 34 wpc, the ssRNA load was estimated at Ct 38.1 ± 1.8, while all the samples at 65 wpc were negative (Figure 2).

In the muscle tissue, the viral load peaked at 2 wpc and steadily decreased until 65 wpc (Figure 2). Significant differences in viral load observed between 12 and 18 wpc (*p* < 0.05), and between 34 and 44 wpc (*p* < 0.0001). Similar to the heart, the ssRNA load was very low at 34 wpc (mean Ct 39.1 ± 1.3), and no ssRNA was detected at 65 wpc.

### PRV-1 persists in blood cells and lymphoid organs of Atlantic salmon until 65 wpc

**Figure 3:**
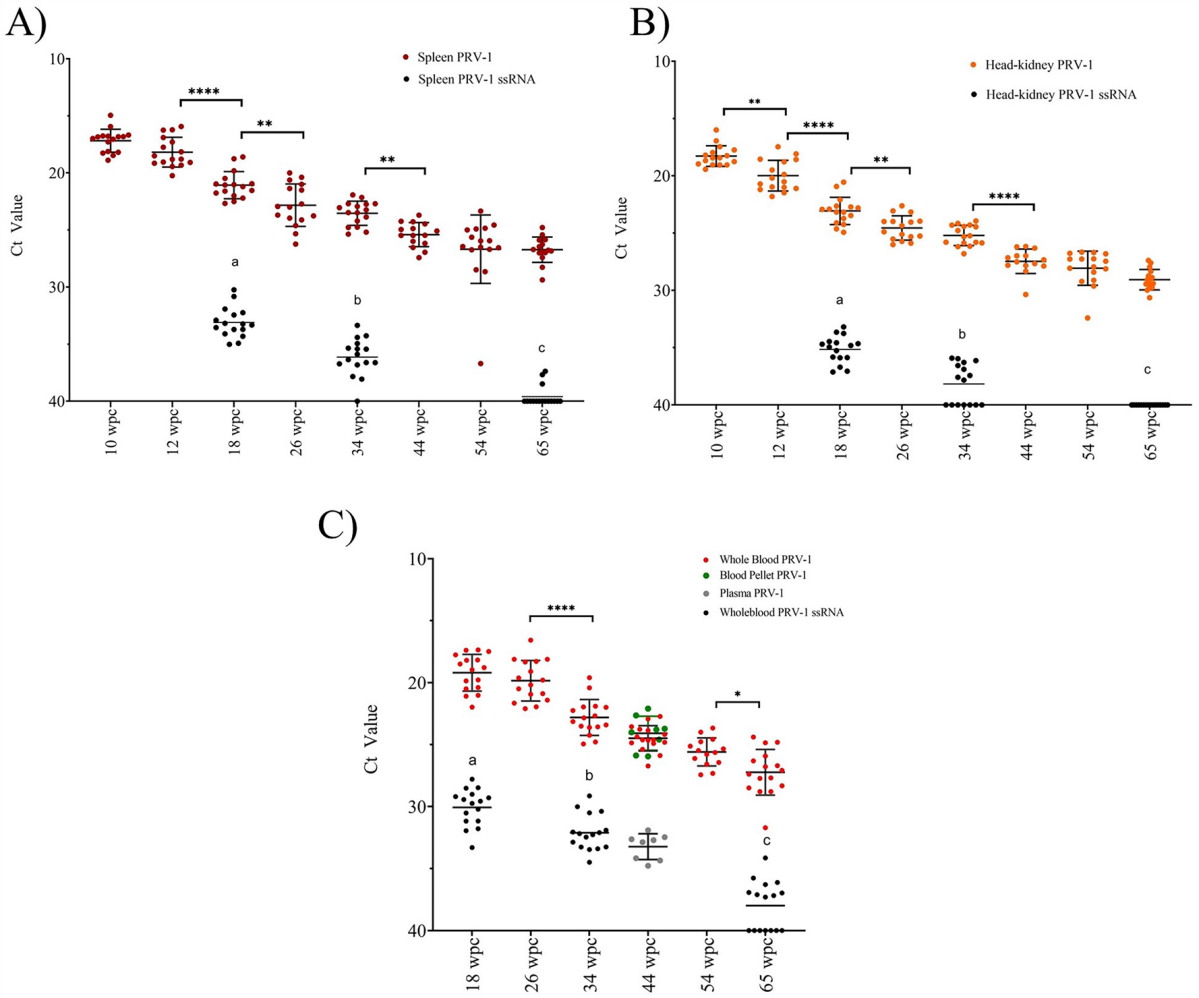
**The viral load of PRV-1 in the longitudinal challenge trial where salmon fry were IP challenged with PRV-1 and followed for 65 weeks.** The PRV-1 viral load in **A** Spleen, **B** Head kidney, and **C** Blood are indicated with red dots. PRV-1 single stranded RNA is shown with black dots. In **C** green dots show the viral load in blood cells and grey dots show viral load in plasma. Statistically significant differences are shown by combined bars and asterisks for total RNA; alphabetical letters for ssRNA. Asterisk (*P < 0.05; **P < 0.01; ***P < 0.001; ****P < 0.0001) indicate significant differences.

Due to the small size of the fry, the blood samples were not taken until 18 wpc. The viral load in whole blood was estimated from 18 wpc onwards, showing a gradual decrease towards 65 wpc (Figure 3). In contrast to heart and muscle, a significant reduction was observed in whole blood between 26 and 34 wpc (Figure 3). At 44 wpc, the viral load in blood fractions (blood pellet and plasma) were also measured. The viral load was higher in the blood cell pellets (24.1 ± 1.4) than in plasma (33.2 ± 1.0). At 65 wpc, the viral load in whole blood was determined to be Ct 27.23 ± 1.8 (Figure 3). In blood, the ssRNA load was significantly lower than the PRV-1 load at all samplings, decreased significantly towards 65 wpc but was still detectable in 9 of 16 fish at 65 wpc (mean Ct 37.99).

Over time, the viral load in the spleen was comparable to the viral load in whole blood. A significant difference in spleen viral load was observed between 12 and 18 wpc (*p* < 0.0001) (Figure 3). The ssRNA load in spleen was estimated to be Ct 35.9 ± 1.3 at 34 wpc and Ct 39.6 ± 0.9 at 65 wpc. The viral load in the head kidney was determined from 10 wpc until 65 wpc, generally having slightly lower viral loads than spleen, but showing a very similar gradual decrease (Figure 3). The ssRNA load in head kidney at 34 wpc was estimated to be Ct 37.16 ± 1.7, and no ssRNA was detected at 65 wpc (Figure 3).

At 65 wpc, head-kidney had a significantly lower viral load compared to spleen and whole blood. Furthermore, the ssRNA load was significantly higher in blood at 34 and 65 wpc compared to the spleen, head kidney, and muscle (Figures 2 and 3).

### Cohabitation experiments showed limited or no PRV-1 transmission

**Figure 4:**
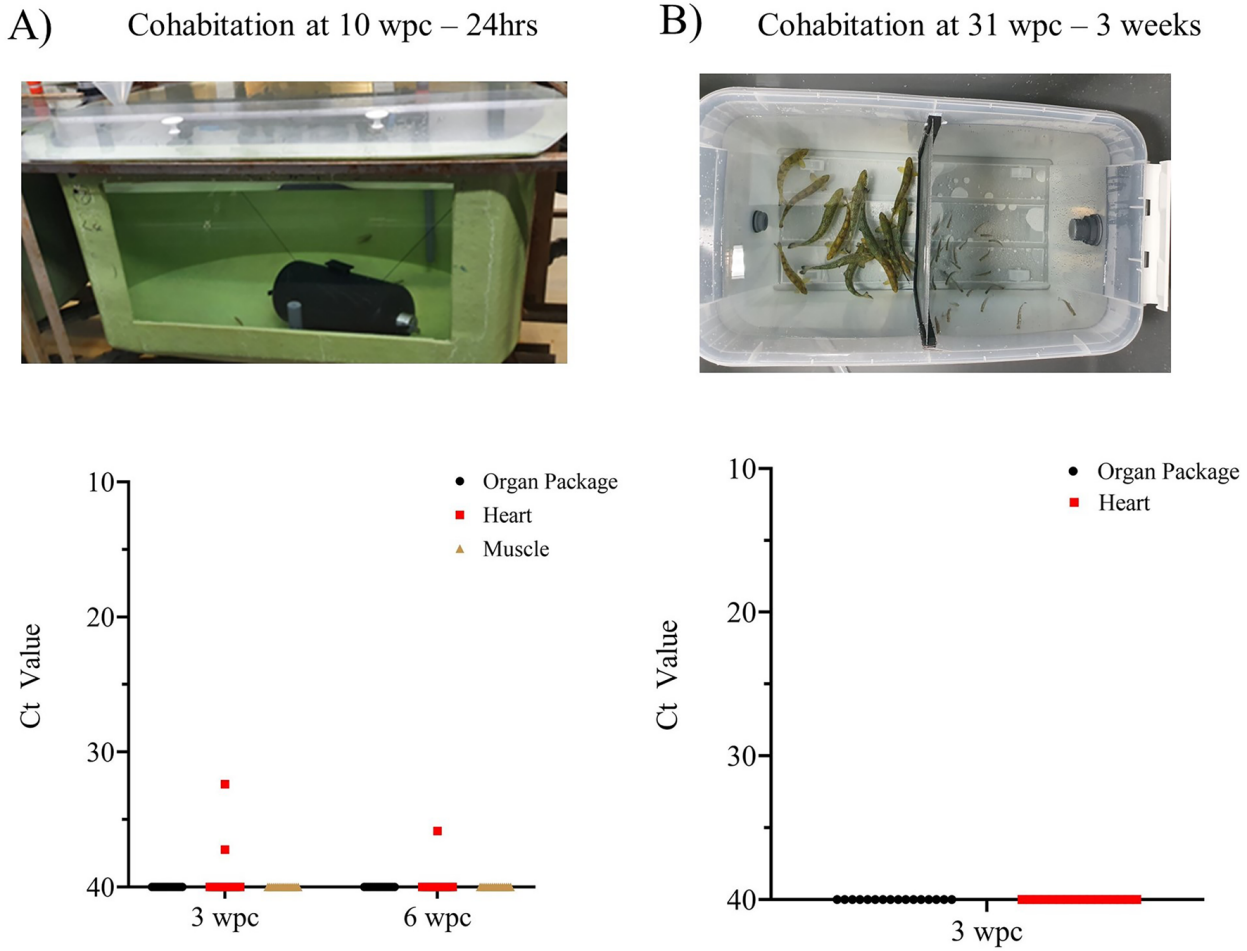
**PRV-1 cohabitation experiments performed at 10 and 31 wpc.** **A** Picture shows the small closed perforated tanks containing the naïve fry that were immersed for 24 h in the 250 L tanks with infected shedder fish in the experiment at 10 wpc. The PRV-1 viral load in samples from the cohabitant salmon fry at three and six weeks post-cohabitation challenge is shown below. **B** Picture shows cohabitation between infected shedders (left) and naïve fry (right) separated by a perforated wall (center) at 31 wpc. The PRV-1 viral load in organ package and heart from the cohabitant salmon fry at three weeks post-cohabitation challenge is shown below. The PRV-1 viral load was measured in the organ package (black dots), heart (red squares), and muscle (gold triangles).

Cohabitation experiments were conducted at 10 and 31 wpc with naïve fry. At 10 wpc, the viral load in the tissues of the PRV-1 infected shedders were measured to be: heart (mean Ct 20.52 ± 1.1), and spleen (mean Ct 17.18 ± 1.0). In the 10 wpc cohabitation experiment, two cohabitant fish hearts were found to be PRV-1 positive with low viral load (Ct 32.39, 37.22) at three weeks post-cohabitation challenge and one cohabitant fish heart was PRV-1 positive with a Ct of 35.85 at six weeks post-cohabitation (Figure 4A). PRV-1 was not detected in the muscle and organ package samples at 3 and 6 wpc.

In the cohabitation experiment conducted at 31 wpc, the viral load in the hearts and spleen of shedder fish were between Ct 25.20 ± 1.95–26.23 ± 1.30 and Ct 22.83 ± 1.86–23.54 ± 1.06, respectively. PRV-1 was not detected in any of the naïve cohabitant salmon fry at 3 wpc (Figure 4B).

### Serial sampling showed decrease in PRV-1 load in blood with time

**Figure 5:**
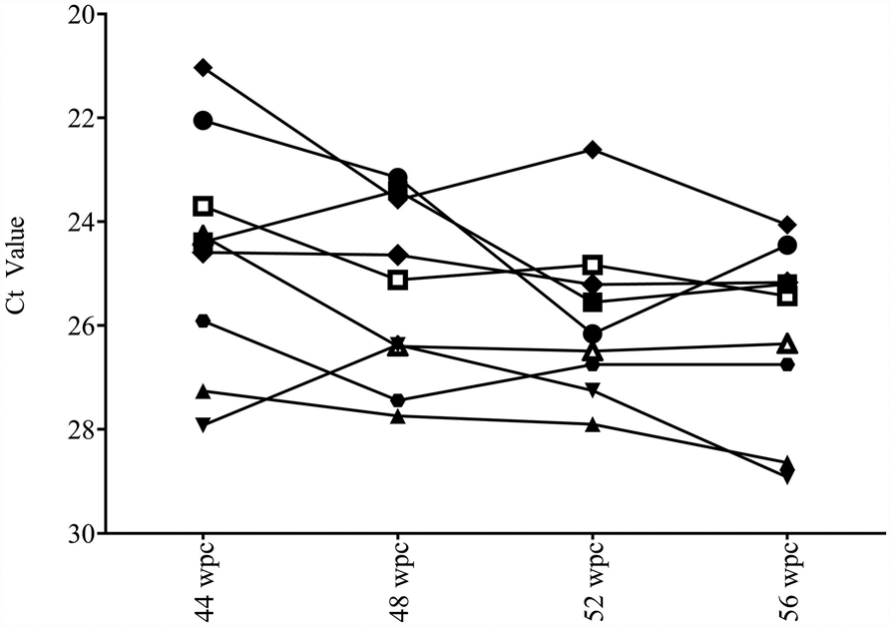
**PRV-1 viral load in blood of PIT tagged, serially sampled individual, infected fish from 44 to 56 wpc.** Blood was sampled from the caudal vein of 9 fish at 44, 48, 52 and 56 wpc and analyzed by qPCR.

The viral load in the blood collected by repeated sampling of individual PIT tagged fish was estimated by RT-qPCR. At 44 wpc, the viral load of individual fish ranged from Ct 21.03 to 27.92, and at 56 wpc, Ct 24.06–28.92. There was an overall reduction in viral load of all the 9 fish from 44 to 56 wpc (Figure 5).

### Stress exposure does not affect the viral load/shedding of PRV-1 infected fish

**Figure 6:**
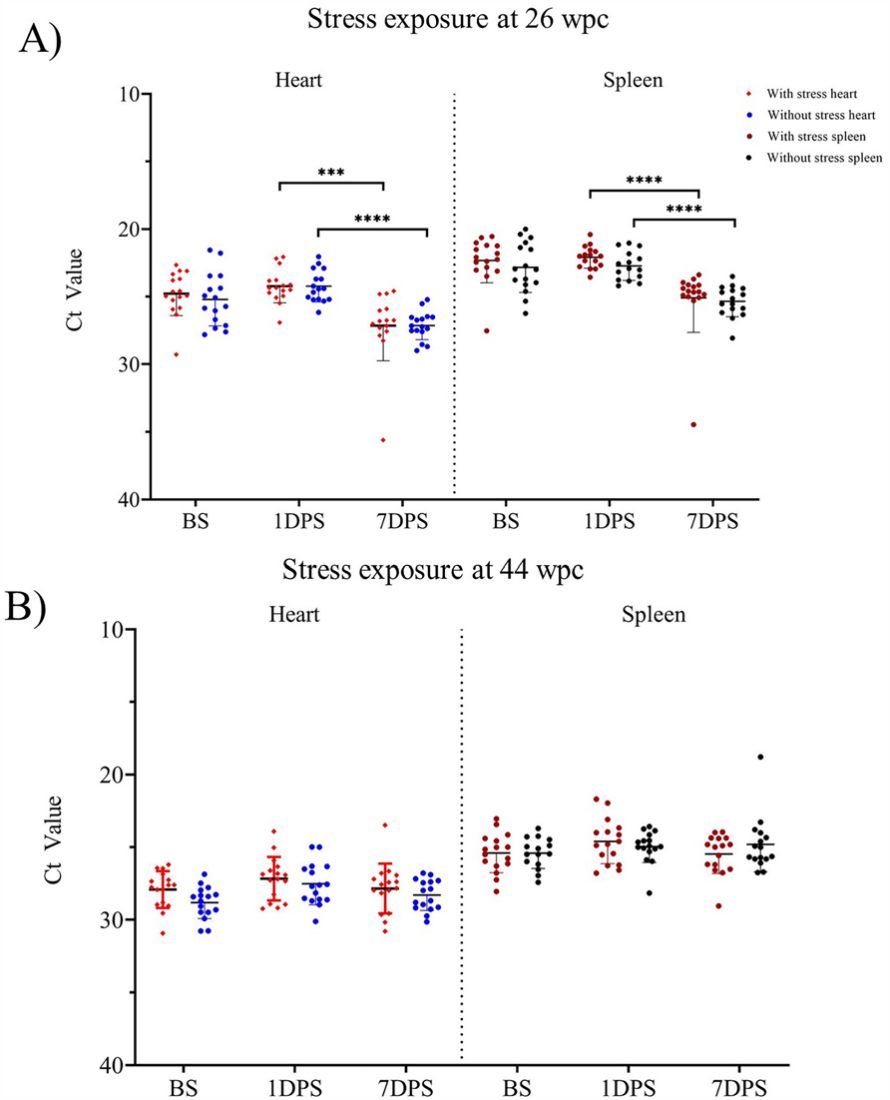
**The effect of stress on PRV-1 viral load.** At two time points (26 and 44 wpc) infected and control fish were subjected to a 7-day stress exposure protocol, after which fish were sampled at different time points post-stress. PRV-1 viral load in stressed and non-stressed fish groups sampled before stress exposure (BS) and 1 and 7 days post-stress (dps) at **A** 26 wpc and **B** 44 wpc. The PRV-1 viral load was measured in the hearts and spleens of stressed and control fish. Viral load in the hearts of stressed fish (red diamonds); viral load in the hearts of control fish (blue dots); viral load in the spleens of stressed fish (red dots); viral load in the spleens of control fish (black dots). Statistically significant differences are shown by combined bars and asterisks. Asterisk (***P < 0.001; ****P < 0.0001) indicate significant differences.

PRV-1 infected fish were exposed to stress for seven days at 26 and 44 wpc. The viral load was estimated in heart and spleen of fish sampled before stress (BS) and 1- and 7-days post-stress (dps). The results in both stress exposure experiments were similar. No significant difference in PRV-1 load between stressed and non-stressed fish was observed at any time point or in any tissue. The mean viral load in the hearts of stressed fish decreased significantly at 7 dps. Similarly, in spleen the viral load before stress was Ct 22.32 ± 1.6 and decreased significantly 7 dps Ct 25.09 ± 2.5 (Figure 6A). A similar trend was observed in the fish groups which were not exposed to stress. The six-week cohabitation experiment with stressed PRV-1 infected shedder fish at 31 wpc did not result in detectable PRV-1 infection in naïve fry (Additional file 2).

### PRV-1 infection causes transient increase in innate and adaptive antiviral immune responses

**Figure 7:**
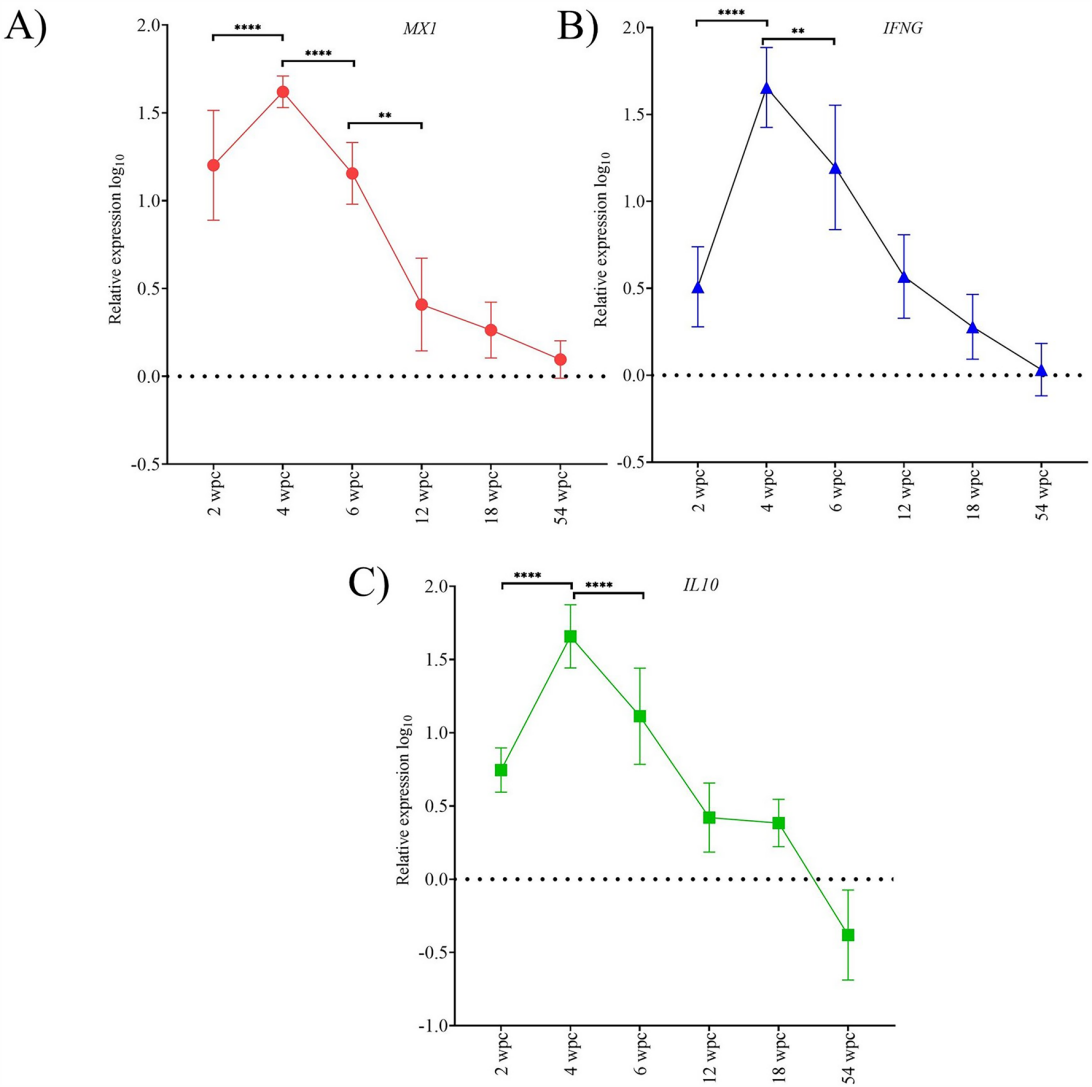
**Innate immune gene expression during the first 54 weeks of the longitudinal challenge trial.** Expression of innate antiviral immune genes *MX1*, *IL10*, and *IFNG* in PRV-1 infected heart tissue (*n* = 8) determined by RT-qPCR. Relative fold changes (mean ± standard deviation) were determined by normalizing against the control fish group sampled at the same time point, data presented as log_10_ transformed. Statistically significant differences are shown by combined bars and asterisks. Asterisk (*P < 0.05; **P < 0.01; ***P < 0.001; ****P < 0.0001) indicate significant differences.

**Figure 8:**
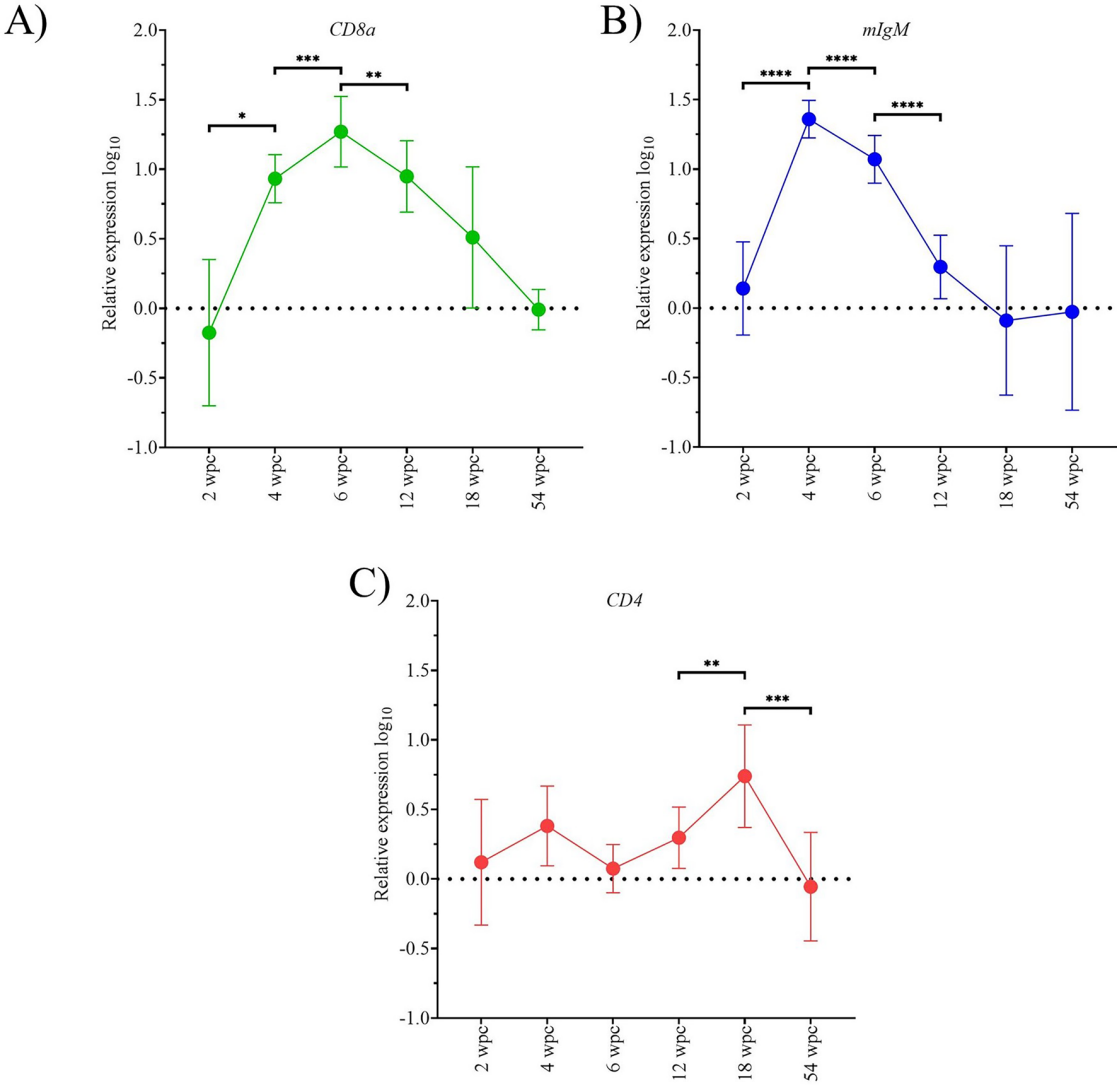
**Adaptive immune gene expression during the first 54 weeks of the longitudinal challenge trial.** Adaptive immune gene expression in PRV-1 infected heart tissue (*n* = 8) determined by RT-qPCR. Relative fold changes (mean ± standard deviation) were determined by normalizing against the control fish group sampled at the same time point, data presented as log_10_ transformed A) *CD8a*, B) *mIgM*, and C) *CD4*. Statistically significant differences are shown by combined bars and asterisks. Asterisk (*P < 0.05; **P < 0.01; ***P < 0.001; ****P < 0.0001) indicate significant differences.

The innate antiviral immune responses in the heart were determined for PRV-1 infected and control fish at 2, 4, 6, 12, 18, and 54 wpc (Figure 7). The expression level of three innate immune genes (*MX1*, *IL10* and *IFNG*) followed a similar pattern. The immune gene transcripts increased significantly from 2 wpc and peaked at 4 wpc, with *MX1*, *IL10*, and *IFNG* showing a 42-, 50-, and 51-fold increase compared to control fish, respectively. The expression then decreased significantly reaching basal levels at 12 wpc. The transcript levels of the adaptive immune genes, *CD4*, *CD8a*, and *mIgM* were also determined in the heart of PRV-1 infected and control fish at these sampling points (Figure 8). The *CD8a* response was significantly increased at 4 wpc, peaking at 6 wpc, coinciding with histopathology, and then decreased significantly (*p* < 0.0002) afterwards. A similar pattern was observed for *mIgM* transcripts, expression of which increased significantly, peaking at 4 wpc and significantly decreasing afterwards. The change in *CD4* transcripts was much less prominent and was only increased significantly at 18 wpc, after which it decreased significantly towards 54 wpc.

### Heart and muscle pathology is resolved after acute inflammation

**Figure 9:**
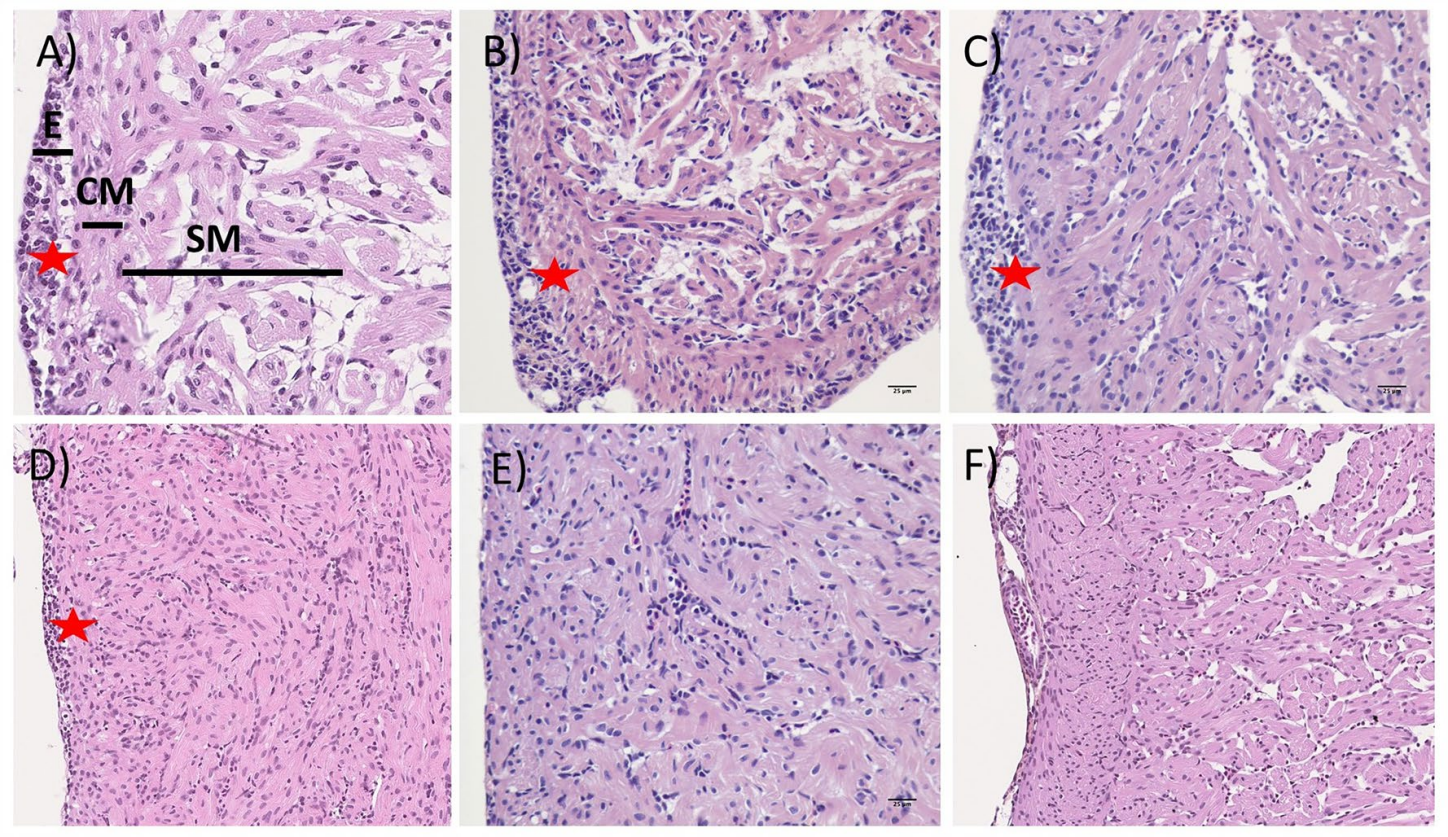
**Heart histopathology during the first 18 weeks of the longitudinal challenge trial.** Histopathological changes in heart of PRV-1 infected fish at **A** onset of inflammation with epicarditis in few places at 4 wpc, **B** both epicarditis and myocarditis in spongy compact and spongy myocardium throughout the heart at 6 wpc, and **C** 8 wpc, **D** Epicarditis and myocarditis observed in some of the fish at 10 wpc, **E** mild inflammation at 12 wpc, and **F** All the fish in the PRV-1 infected groups resolved inflammation at 18 wpc. E- epicardium, CM- compact myocardium, SM- spongy myocardium, and red stars highlight epicarditis.

**Figure 10:**
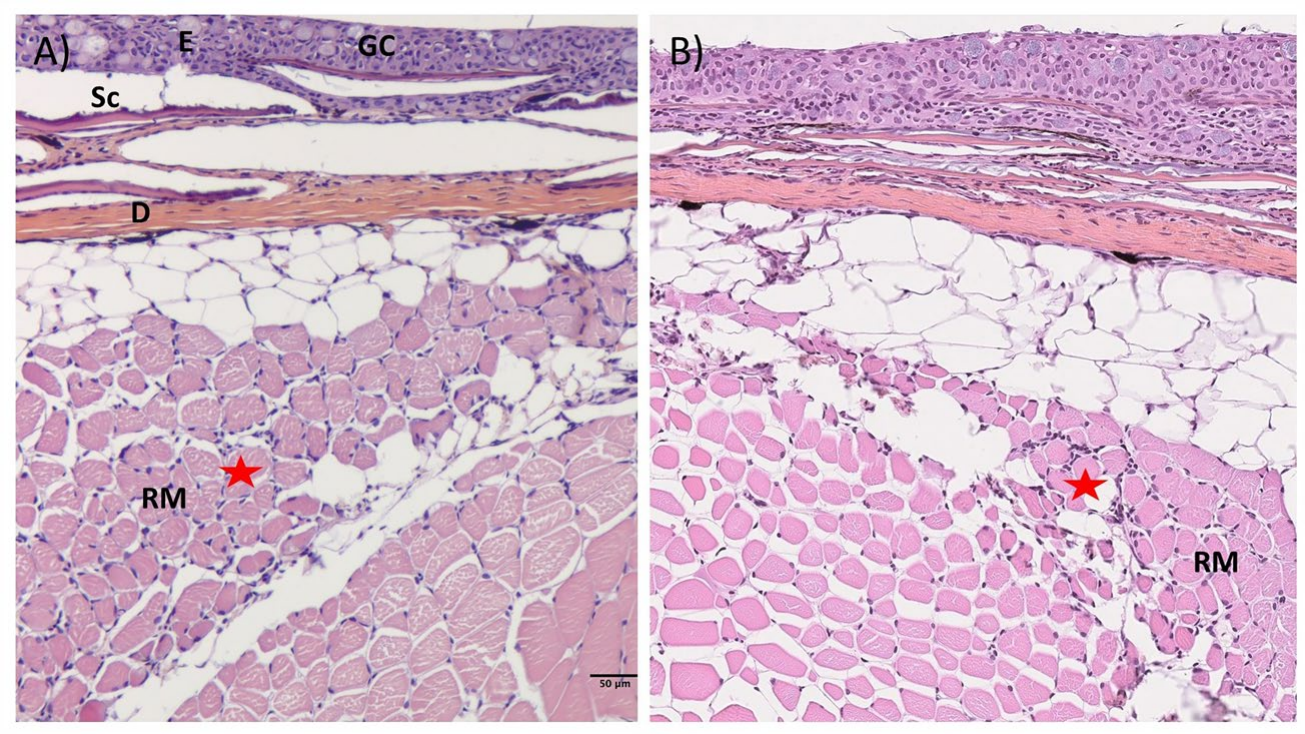
**Muscle histopathology during the first 10 weeks of the longitudinal challenge trial.** Histopathological changes in muscle of PRV-1 infected fish at **A** inflammation observed in red muscle at 8wpc, and **B** 10wpc. E- epidermis, Sc- scale, GC- goblet cells, D- dermis, RM- red muscle, and red start highlights inflammation in red muscle tissues.

PRV-1 infection in Atlantic salmon fry resulted in histopathological changes in heart, including epicarditis and myocarditis in both compact and spongy myocardium (Figure 9). The inflammation began at 4 wpc with mild epicarditis and reached its peak at 6 and 8 wpc but decreased to mild inflammation at 10 wpc. By 12 wpc, most of the inflammation in both epicardium and myocardium was resolved. In the muscle, infiltration of inflammatory cells was observed mainly in red muscle at 8 and 10 wpc (Figure 10). However, no inflammation was observed in either red or white muscle after 12 weeks.

## Discussion

Piscine orthoreovirus-1 (PRV-1) causes the disease heart and skeletal muscle inflammation (HSMI) in farmed Atlantic salmon. In Norway, PRV-1 is considered widespread, however, the incidence of HSMI in both freshwater pre-smolts and seawater post-smolts is uncertain. HSMI outbreaks are most common in seawater, and can occur as early as 14 days but also up to 5 months after seawater transfer [36]. Clinical or subclinical disease outbreaks can last for weeks or months and PRV infections can be detected for a longer time as there is likely continuous shedding and transmission among individual fish. In a longitudinal study, PRV-1 was detected for more than 8 months after the first detection with a general decrease in infection prevalence and viral load over time [37]. However, the viral kinetics and disease development in freshwater pre-smolt stages are not known. Here, we describe the PRV-1 infection in fry and parr stages of Atlantic salmon over the course of 65 weeks.

The viral load peaked at 2–4 weeks post-challenge and decreased subsequently during the course of 65 weeks. The PRV-1 virus load decreased gradually but the virus persisted in blood cells, heart, muscle, spleen and head kidney until at least 65 weeks. Based on previous studies [18, 38], and the results from the current study, the following stages can be suggested for PRV-1 replication, pathogenesis in Atlantic salmon. An acute phase that stretches from 0 to 12 wpc. During this period our observations encompassed the following events: peak of viral load, onset of disease/pathology, resolution of inflammation in heart, muscle, and induction of immune response. Independent studies have reported viral shedding, clearance of viral factory proteins in blood [11], and production of antibodies transpiring within this acute phase [18]. The acute phase can be further sub-divided into early acute phase (0–4 wpc – peak of viral infection in blood, heart, and muscle) and late acute phase (4- 12 wpc—peak of pathology and resolution, clearance in heart and muscle [14]). Following the acute phase, the persistent phase (12 wpc onwards) is characterized by decreasing replication and little or no transmission, resolved heart pathology, and a decrease in the immune response to basal levels.

Previous PRV-1 challenge studies have shown that plasma viremia coincides with the peak of viral load in erythrocytes [2, 38]. Subsequently, the viral load in plasma decreased and the viral RNA could be detected at low levels until 18 weeks post challenge [38]. In this study, we detected viral RNA in plasma at low levels during the persistent phase of infection (44 wpc) while levels of PRV-1 RNA were high in both whole blood and blood cell pellet, suggesting that the virus is primarily present in infected blood cells which is highly dominated by erythrocytes.

PRV is a double stranded RNA virus that transcribes single stranded RNA (ssRNA) during active viral replication for protein synthesis and genome replication. In RT-qPCR, the genomic RNA (gRNA) and ssRNA can be differentially quantified by adding a denaturing step before cDNA synthesis [10]. For PRV-1 it has been shown that the proportion of ssRNA to total RNA is high during acute active viral replication, but decreases from 90% at 4 wpc to less than 0.1–1% by 7–8 wpc [10]. In a previous PRV-1 challenge study, ssRNA was detected at high levels in blood and kidney until 18 wpc [38]. In the present study, the ssRNA level in heart peaked along with the viral load during acute phase (2–4 wpc) and decreased significantly at 6 wpc. There are low but detectable levels of ssRNA at 18 and 34 wpc in all the tissues. At 65 wpc, only blood (in 9 of 16 fish) and spleen (3 of 16) had detectable ssRNA, and the ssRNA level was consistently higher in blood than other tissues. In contrast, a previous study reported that the ssRNA level at 18 wpc was higher in head kidney than blood cells [38]. The absence of detectable ssRNA in head kidney, heart and muscle at 65 wpc indicates the existence of non-transcribing dormant/defective virus during the persistent phase, challenging the idea of viral persistence sustained via amplification in erythroid progenitor cells or other undisclosed sites of virus replication [38].

In this study, our findings demonstrate that PRV-1 RNA can persist in infected blood cells for over 65 weeks (more than 455 days). This is noteworthy considering that the life span of the erythrocytes is between 80 and 500 days [39]. The development in mean Ct values observed between 18 and 65 wpc (19.20 at 18 wpc and 27.23 at 65 wpc) suggests a 260-fold reduction in viral load. Interestingly, this is paralleled by a very similar reduction in the ssRNA load of 240-fold (Ct 30.07 at 18 wpc and 37.99 at 65 wpc) during the same period. Further, the ratio between viral load and ssRNA load is relatively constant (1870-, 635- and 1734-fold at 18, 34 and 65 wpc, respectively) during the 18 to 65 wpc period. This observation is compatible with a scenario where a finite number of PRV-1 infected blood cells, having a fixed ratio of viral to ssRNA load, are gradually removed from the circulation with little or no replenishment of newly infected cells. In extension thereof, a fixed ratio of viral to ssRNA load would suggest that many/most of the infected blood cells retain some level of viral replication/activity until removed. The fact that PRV-1 infected erythrocytes, possibly with a level of viral activity, can stay in circulation for extended periods, suggests that PRV-1 applies specific measures to avoid the effect of the salmon immune system.

The gene expression data in this study show both activation of innate immune genes and influx of CD8 cytotoxic T-cells and mIgM positive B-cells in the infected heart. Given that inflammation and histopathology is then resolved, and the immune activity returns to basal levels in the heart, it seems reasonable to conclude that immunity is efficient in clearing PRV-1 in this tissue. It is therefore remarkable that the immune system cannot clear the circulation of infected erythrocytes even over extended periods. PRV-1 infection in erythrocytes upregulates interferons and interferon regulatory proteins, as well as antiviral proteins such as Mx, viperin, PKR, and ISG-15 which induce an antiviral state in infected and neighboring cells [40], but this seems incapable of halting PRV-1 from spreading to > 50% of the RBC [12]. With the advent of circulating PRV-1 specific antibodies, viral transmission between erythrocytes (and other cell populations) is likely reduced or blocked, but the cellular immune system is not capable of lysing/removing infected erythrocytes. The failure to eliminate infected cells could be due to immune evasion, downregulation/inactivation of cellular immune protection mechanisms, or exhaustion/tolerance of T cells. The inability to specifically remove PRV-1 infected erythrocytes from the circulation could be a mechanism for viral persistence, and the limiting lifespan of erythrocytes would explain the gradual decay of this persistence.

Despite the high viral load in PRV-1 infected fish at 10 wpc and 34 wpc, only very few naïve fry cohabitating with these fish were infected. At 10 wpc, two cohabitant fish at 3 weeks post cohabitation challenge and one cohabitant fish at 6 weeks tested positive for PRV-1. But at 31 wpc, none of the naïve cohabitant fry were infected. Given the relative high susceptibility of salmon fry [41], the lack of transmission is likely due to very low levels of infectious virus in the water, suggesting limited or no shedding of infectious virus from the infected fish. Since PRV-1 is not able to be grown in cell culture, the minimal infectious dose (MID) required to establish primary infection is not known. A previous study has shown that the PRV-1 low virulent genotype can persist in Atlantic salmon for 59 weeks in heart and kidney tissues and that PRV-1 persistent fish at 41 wpc failed to infect age matched naïve cohabitants [16].

In another PRV-1 cohabitation study, cohabitation of fish with shedders were done right from the beginning, and typically, infection among cohabitants was observed after four weeks post-cohabitation [42]. Our results suggest that the shedding of PRV-1 is insufficient to establish 100% infection in cohabitants after 10 wpc. The early acute phase of infection appears to be critical for horizontal transmission of PRV-1. However, the precise timing and duration of the shedding window during the acute phase require further investigation. The stress exposure used in the study did not result in resurgence of shedding or an increase in viral load. However, other stress factors present in farming conditions such as sea lice treatment, handling/moving fish during persistent phase of infection should be tested for the risk of resurgence/transmission.

During the acute phase, weight differences were observed between control fish and PRV-1 infected fish at 8, 10, and 12 wpc (Additional file 3). The spatiotemporal viral load analysis of PRV-1 infected fish over a 65 week period, suggests that blood/spleen are the most suitable tissues for determining the viral load. In addition, qPCR with and without denaturation of RNA can be employed to differentiate between phases of infection. This approach can be helpful in identifying the timing of infection in escapees or wild fish.

During the acute phase of PRV-1 infection, innate antiviral immune response genes, such as *MX1*, *IL10*, and *IFNG* were significantly upregulated but had returned to basal levels by 12 wpc. Previous studies have reported differences in the induction of innate immune genes depending on the PRV genotype and host species [16]. Earlier studies support our observations, showing that the innate immune response is upregulated during the acute phase of PRV infection and returned to basal level afterwards [10, 40]. In Atlantic salmon post-smolts, upregulation of *MX1* transcripts following PRV-1 infection has been observed, with higher level of *MX1* response seen until 6 or 10 wpc depending on the challenge method and viral dose, after which it returns to basal levels [2].

Interferon gamma (IFNγ) is mainly secreted by immune cells and belongs to the type II interferon family. Interferons induce the expression of IFN stimulated genes (ISG) and establish inflammation. The *IFNG* response is usually delayed compared to type I interferons [43]. In the present study, the *IFNG* response was low at 2 wpc but peaked at 4 wpc before decreasing after 4 wpc. IL10 is known to function as an anti-inflammatory cytokine in fish. Despite an increase in *IL10* expression following PRV-1 infection, the inflammatory response in the heart was not completely suppressed. Similar expression patterns for both *IFNG* and *IL10* were observed in a previous study following infectious pancreatic necrosis virus (IPNV) infection [44]. All of the innate immune response genes followed a similar kinetics, that also correlated with viral load (data not shown).

The expression of adaptive immune response genes, *mIgM*, *CD4* and *CD8a* was significantly upregulated in heart tissues of PRV-1 infected fish, indicating both B/T cell mediated immune responses and influx of B and T cells to the site of infection/inflammation. The *CD8a* response showed a significant increase at 4 wpc, peaked at 6 wpc, and subsequently decreased. Consistent with earlier studies, the *CD8a* response correlated with heart pathology. Over the course of our study, the innate and adaptive immune responses did return to basal levels, indicating that persistent PRV-1 infection did not continue to stimulate the immune response indefinitely.

In conclusion, the results of this study indicate that PRV-1 infection in Atlantic salmon fry establishes a persistent infection, with a long-term presence in the host’s in blood cells and lymphoid organs that lasts for at least 65 weeks. Despite a very high viral load in blood, PRV-1 positive fish were not infectious in cohabitation trials at 31 wpc and only to a minor degree at 10 wpc. The stress exposure during the persistent phase did not increase the viral load or exacerbate disease progression or shedding. However, it would be valuable to investigate the effects of additional stress, such as smoltification, superinfection or sea lice treatment and its effect on virus recurrence and disease development. Despite this, our findings provide important insights into the persistence of PRV-1 infection in Atlantic salmon fry, parr, and pre-smolts. However, further research is needed to fully understand the mechanisms of PRV-1 persistence.

### Supplementary Information

**Additional file 1: PRV-1 viral load in the blood cells and plasma of PRV-1 infected fish in vivo propagation.** The PRV-1 viral load was estimated every week and the blood was harvested at 4 wpc during peak of the infection.

**Additional file 2: PRV-1 viral load in non-contact cohabitant salmon fry at 31 wpc.** Cohabitation with naïve fry was done for 6 weeks in a tank divided between PRV-1 shedders parr and naïve fry like shown in Figure 3B.

**Additional file 3: Weight (in gram) of PRV-1 and control fish in the longitudinal challenge trial** Atlantic salmon fry were IP challenged with PRV-1 and followed for 65 weeks A) 2-12 wpc and B) 18-65 wpc.

## Data Availability

Data beyond the results presented in the paper is available upon request to corresponding author.
