# Supplementary figures and images for: Long-term persistence of piscine orthoreovirus-1 (PRV-1) infection during the pre-smolt stages of Atlantic salmon in freshwater
Source: Vet Res. 2023 Aug 29;54:69. doi: 10.1186/s13567-023-01201-w (PMC10463814; doi:10.1186/s13567-023-01201-w)

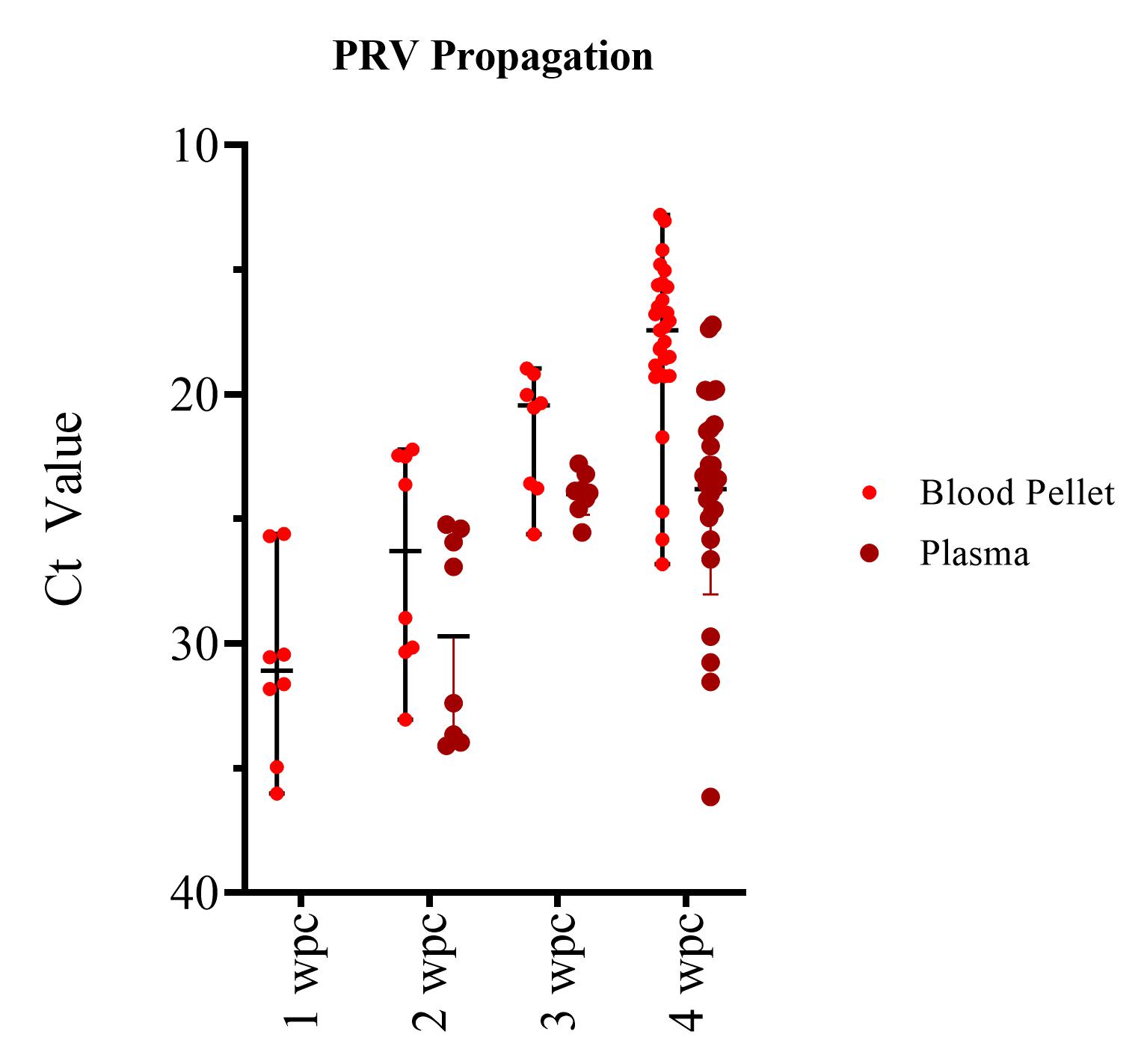

Supplement: Supplementary file 1 — Additional file 1: PRV-1 viral load in the blood cells and plasma of PRV-1 infected fish in vivo propagation. The PRV-1 viral load was estimated every week and the blood was harvested at 4 wpc during peak of the infection. [file 13567_2023_1201_MOESM1_ESM.jpg]

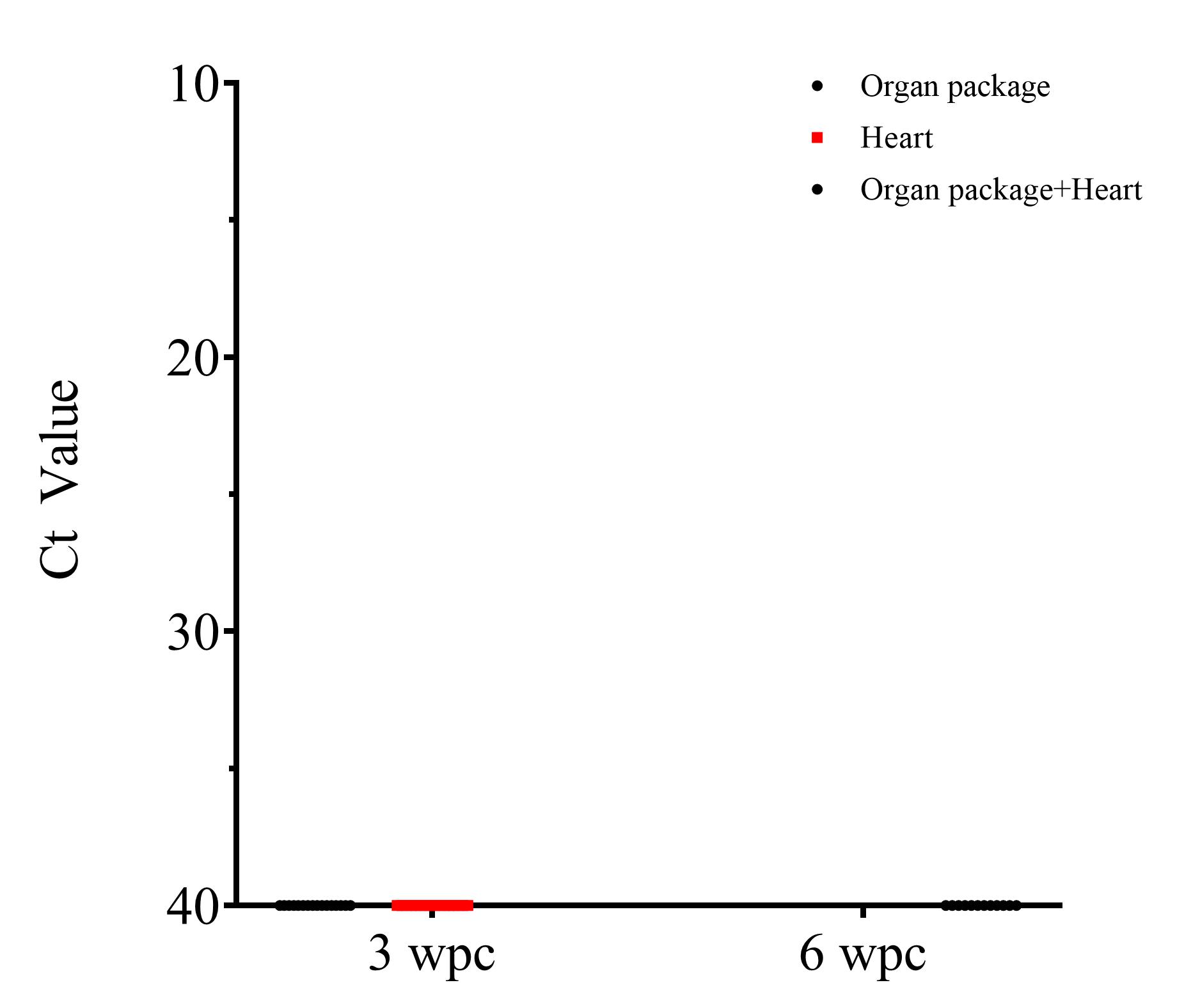

Supplement: Supplementary file 2 — Additional file 2: PRV-1 viral load in non-contact cohabitant salmon fry at 31 wpc. Cohabitation with naïve fry was done for 6 weeks in a tank divided between PRV-1 shedders parr and naïve fry like shown in Figure 3B. [file 13567_2023_1201_MOESM2_ESM.jpg]

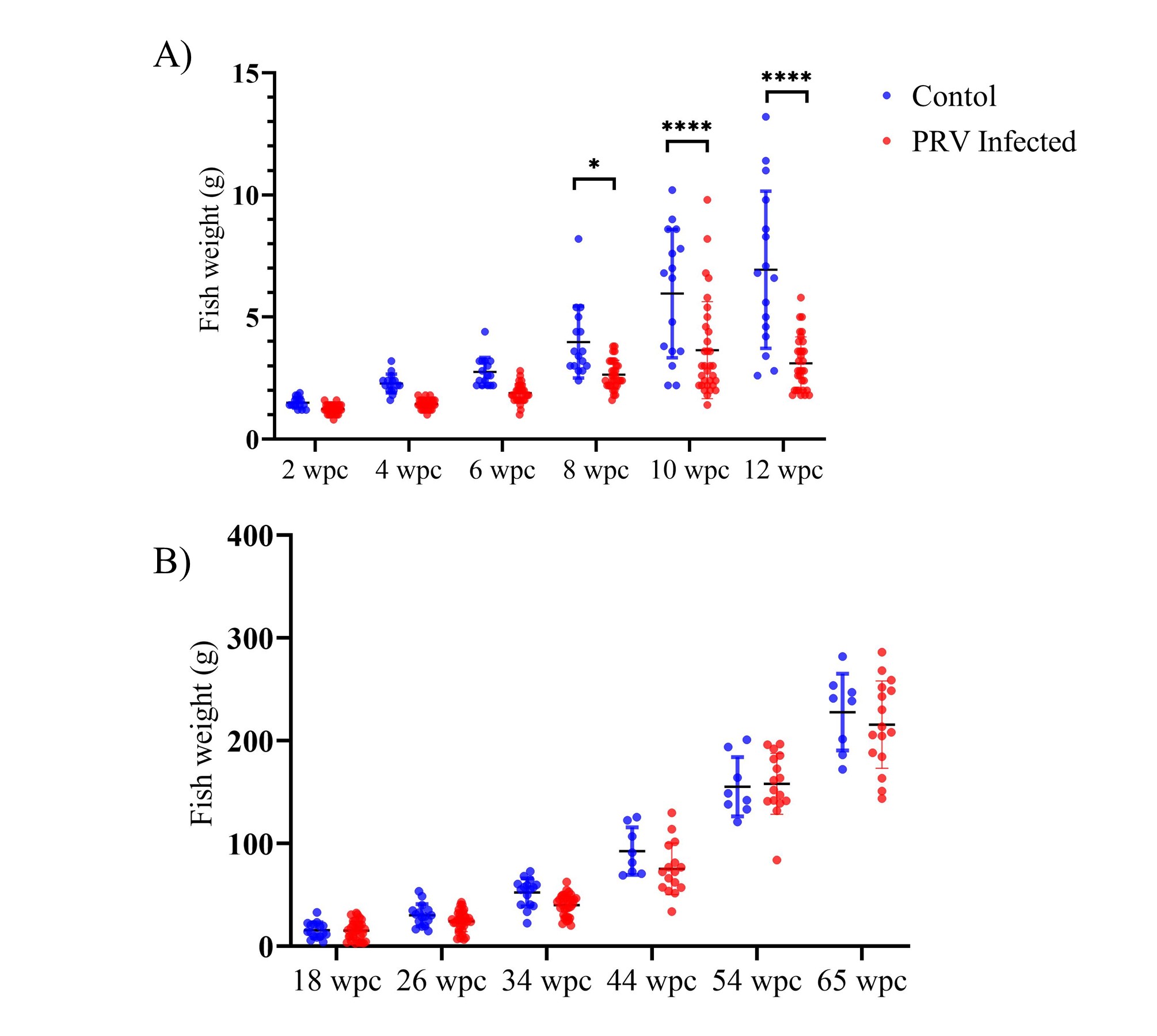

Supplement: Supplementary file 3 — Additional file 3: Weight (in gram) of PRV-1 and control fish in the longitudinal challenge trial Atlantic salmon fry were IP challenged with PRV-1 and followed for 65 weeks A) 2-12 wpc and B) 18-65 wpc. [file 13567_2023_1201_MOESM3_ESM.jpg]
